# Supplementary material for: Effect of restricting bedtime mobile phone use on sleep, arousal, mood, and working memory: A randomized pilot trial
Source: PLoS One. 2020 Feb 10;15(2):e0228756. doi: 10.1371/journal.pone.0228756 (PMC7010281; doi:10.1371/journal.pone.0228756)
Supplement: S2 File — (DOCX) [file pone.0228756.s002.docx]

**【一般情况】**

请在相应位置填写或在符合您实际情况选项前的“**□**”里打“√”

**1、姓名：**  **专业（年级）：**

**2、性别：□**男 **□**女 **3、年龄：** 岁

**4、身高：** 厘米（cm） **体重：** 千克（kg）

**5、教育程度：□**未上过学 **□**小学 **□**中学 **□**大专 **□**本科及以上

**6、婚姻状况：□**在婚 **□**未婚 **□**离婚 **□**丧偶

**7、物质使用情况（您是否有以下物质使用情况，若有，请在方框中打“√”，并注明使用情况）：**

**（1）咖啡饮用情况：**

**□**很少或从不饮用 **□**偶尔饮用 **□**经常饮用或几乎每天  **（2）茶(量/时间)：**

**□**很少或从不饮用 **□**偶尔饮用 **□**经常饮用或几乎每天

**（3）烟(量/时间)：**

**□**很少或从不吸烟 **□**偶尔吸烟 **□**经常吸烟或几乎每天

**（4）酒(量/时间)：**

**□**很少或从不饮用 **□**偶尔饮用 **□**经常饮用或几乎每天

**8、平均每天静坐多少时间： 小时**

**9、平时的运动情况**：**（平均一周运动 次，每次运动多少时间 小时）**

**□**很少运动 **□**有时运动 **□**经常运动

**10、平均每天使用手机、pad等电子产品多少时间：**  **小时**

**11、使用电子产品用于游戏 小时；娱乐聊天 小时；工作学习 小时**

1**2、平均每天睡前使用手机、pad等电子产品多少时间:** **小时**

**一．问卷一**

下面一些问题是源于您**最近1个月**的睡眠状况。请回答下列问题:

1.**近1个月**，晚上上床睡觉通常是 点钟；.从上床到入睡通常需要 分钟；通常早上 点起床；每夜通常实际睡眠 小时(不等于卧床时间)。

2.对下列问题请选择**1个**最适合您的答案。**近1个月**，因下列情况影响睡眠而烦恼

| (1)入睡困难(不能在30分钟内入睡) | 无 | <1次/周 | 1,2次/周 | ≥3次/周 |
| --- | --- | --- | --- | --- |
| (2)夜间易醒或早醒 | 无 | <1次/周 | 1,2次/周 | ≥3次/周 |
| (3)夜间上厕所 | 无 | <1次/周 | 1,2次/周 | ≥3次/周 |
| (4)出现呼吸不畅 | 无 | <1次/周 | 1,2次/周 | ≥3次/周 |
| (5)咳嗽或鼾声高 | 无 | <1次/周 | 1,2次/周 | ≥3次/周 |
| (6)感觉冷 | 无 | <1次/周 | 1,2次/周 | ≥3次/周 |
| (7)感觉热 | 无 | <1次/周 | 1,2次/周 | ≥3次/周 |
| (8)做噩梦 | 无 | <1次/周 | 1,2次/周 | ≥3次/周 |
| (9)疼痛不适 | 无 | <1次/周 | 1,2次/周 | ≥3次/周 |
| (10)其他影响睡眠的事情 | 无 | <1次/周 | 1,2次/周 | ≥3次/周 |

3.近1个月，总的来说，您认为自己的睡眠质量

A.很好; B.较好; C.较差; D.很差

4.近1个月，您用药物催眠的情况

A.无; B.<1次/周; C.1,2次/周; D.≥3次/周

5.近1个月，您常感到困倦吗

A.无; B.<1次/周; C.1,2次/周; D.≥3次/周

6.近1个月，您做事情的精力不足吗

A.没有; B.偶尔有; C.有时有; D.经常有

7对你当前睡眠模式的满意度：

A.很满意 B.满意 C.一般 D.不满意 E.很不满意

8你认为你的睡眠问题在多大程度上干扰了你的日间功能(如日间疲劳、处理工作和日常事务的能力、注意力、记忆力、情绪等)：

A.没有干扰 B.轻微 C.有些 D.较多 E.很多干扰

9与其他人相比，你的失眠问题对你的生活质量有多大程度的影响或损害：

A.没有 B.一点 C.有些 D.较多 E.很多

10.你对自己当前睡眠问题有多大程度的焦虑和烦扰：

A.没有 B.一点 C.有些 D.较多 E.很多

11.你平时做梦情况：A.没有; B.偶尔有; C.有时有; D.经常有

12.平时是否有午睡的习惯：1.是 2.否 如有，午睡一般睡多久： 小时

**二．问卷二**

请仔细阅读每一题，然后根据您**近期的入睡前情况**，请选择相应的程度

|  | **无** | **很少** | **一般** | **经常** | **总是** |
| --- | --- | --- | --- | --- | --- |
| 1.心慌、气紧，精神紧张 | 1 | 2 | 3 | 4 | 5 |
| 2.颤抖、肌肉紧张不适 | 1 | 2 | 3 | 4 | 5 |
| 3.气短或者呼吸困难 | 1 | 2 | 3 | 4 | 5 |
| 4.感觉全身肌肉紧绷绷的 | 1 | 2 | 3 | 4 | 5 |
| 5.手、脚或全身冰凉感 | 1 | 2 | 3 | 4 | 5 |
| 6.胃部不适(恶心或胃灼热感等) | 1 | 2 | 3 | 4 | 5 |
| 7.手掌或全身出汗 | 1 | 2 | 3 | 4 | 5 |
| 8.口干、喉咙干 | 1 | 2 | 3 | 4 | 5 |
| 9.担心睡着 | 1 | 2 | 3 | 4 | 5 |
| 10.回顾或思考白天的事情 | 1 | 2 | 3 | 4 | 5 |
| 11.沮丧或焦虑的想法 | 1 | 2 | 3 | 4 | 5 |
| 12.担心睡眠以外的问题 | 1 | 2 | 3 | 4 | 5 |
| 13.思维敏捷、活跃 | 1 | 2 | 3 | 4 | 5 |
| 14.不能停止思想 | 1 | 2 | 3 | 4 | 5 |
| 15.头脑异常活跃 | 1 | 2 | 3 | 4 | 5 |
| 16因噪音或其他声音而烦躁(钟表声、屋内外其他噪音等) | 1 | 2 | 3 | 4 | 5 |

**三．问卷三**请阅读每一个词语并根据你近1个星期的实际情况在相应的答案上画圈。

|  | 几乎没有 | 比较少 | 中等程度 | 比较多 | 极其多 |
| --- | --- | --- | --- | --- | --- |
| 1、感兴趣的 | 1 | 2 | 3 | 4 | 5 |
| 2、心烦的 | 1 | 2 | 3 | 4 | 5 |
| 3、精神活力高的 | 1 | 2 | 3 | 4 | 5 |
| 4、心神不宁的 | 1 | 2 | 3 | 4 | 5 |
| 5、劲头足的 | 1 | 2 | 3 | 4 | 5 |
| 6、内疚的 | 1 | 2 | 3 | 4 | 5 |
| 7、恐惧的 | 1 | 2 | 3 | 4 | 5 |
| 8、敌意的 | 1 | 2 | 3 | 4 | 5 |
| 9、热情的 | 1 | 2 | 3 | 4 | 5 |
| 10、自豪的 | 1 | 2 | 3 | 4 | 5 |
| 11、易怒的 | 1 | 2 | 3 | 4 | 5 |
| 12、警觉性高的 | 1 | 2 | 3 | 4 | 5 |
| 13、害羞的 | 1 | 2 | 3 | 4 | 5 |
| 14、备受鼓舞的 | 1 | 2 | 3 | 4 | 5 |
| 15、紧张的 | 1 | 2 | 3 | 4 | 5 |
| 16、意志坚定的 | 1 | 2 | 3 | 4 | 5 |
| 17、注意力集中的 | 1 | 2 | 3 | 4 | 5 |
| 18、坐立不安的 | 1 | 2 | 3 | 4 | 5 |
| 19、有活力的 | 1 | 2 | 3 | 4 | 5 |
| 20、害怕的 | 1 | 2 | 3 | 4 | 5 |
