# Supplementary material for: Effect of restricting bedtime mobile phone use on sleep, arousal, mood, and working memory: A randomized pilot trial
Source: PLoS One. 2020 Feb 10;15(2):e0228756. doi: 10.1371/journal.pone.0228756 (PMC7010281; doi:10.1371/journal.pone.0228756)
Supplement: Attachment — Submitted filename: Response to Reviewers.docx [file pone.0228756.s003.docx]

Dear Editors and Reviewers:

Thank you for your letter and for the reviewers’ comments concerning our manuscript entitled “Effect of Restricting Bedtime Mobile Phone Use on Sleep, Arousal, Mood and Working memory” (ID: PONE-D-19-19506). Those comments are valuable and very helpful for revising and improving our paper, as well as the important guiding significance to our researches. We are writing to provide this letter to explain, point by point, the details of our revisions in the manuscript and our responses to the reviewer’ s comments as follows. We are very sorry that there are some spelling mistakes. Besides, we have carefully checked through the whole manuscript and corrected some grammar mistakes. We hope the revised paper would meet the requirement from you and the reviewer.

We are looking forward to hearing from you soon.

Kind Regards,

**Reviewer #1:**

**Here are the general comments from the reviewer:** It is confused that several significance marks (* or **) were missing in Table 2 and Figs 2 and 3 according to results (Line 202-246). The writing is poor and tables are ugly.

Response: We are grateful for the suggestion. We have added significance marks in Figures. After re-checking Table 2, we think the significance marks were not missing. Because Table 2 was the results of between-subject analyses of variance, with one between-group factor (intervention vs control group) and one within-subjects/repeated measures factor (baseline vs follow-up). And results of simple effect analyses were displayed in Fig2, Fig3 and Fig4. As for the writing of our manuscript and tables, we have carefully double checked through the whole manuscript and corrected some grammar mistakes according to the requirement.

**Minor comments:**

1)Figure legends are missing (only abbreviations).

We have added figure legends and modified them according to PLOS ONE's style requirements.

2)Line 8 and 10: 1 and 2 should be superscript.

1 and 2 have been superscript.

3)Line 30, 138: “dairy”?

We are very sorry for our negligence of writing. “dairy” in line 30 and 138 have been corrected as “diary”.

4) Line 30-34: rephrase with ‘respectively’.

This sentence was rephrased according to the comment. (line 30-34)

5)Line 82-83: rephrase

Line 82-83 was rephrased as “The inclusion criteria were poor sleep quality (Pittsburgh sleep quality index [PSQI]>5) and a habit of using a mobile phone during bedtime (>30 mins).”

6) Line 94: screen should be screened

“Screen” has been corrected as “screened”.

7) Line 94-99: confused, need rephrase

This sentence was rephrased as “A total of 72 individuals were screened, and 32 were found to be ineligible. Out of these 32 individuals, 23 were ineligible because of the presence of other sleep disturbances, five volunteers were ineligible because of their mobile phone use habit during bedtime or for use for less than 30 minutes prior to time of sleep. Two participants were ineligible because of medication use to help with sleep and another two were ineligible because of a history of depression. After screening, two participants were dropped because they were being deployed in the South China sea for medical treatment.”

8) Line 135: missing original reference of PSQI

We have added the original reference of PSQI according to comments.

9) Line 137-138: Sleep diary data of the four weeks between that were collected or not?

Sleep diary data between four weeks were not collected. Line 137-138 have been rephrased to make it less confused.

10）Line 142: rephrase

“Mobile phone usage time prior to bedtime was defined as the use from 9:00 pm to the time of sleep” were rephrase as “Mobile phone usage time prior to bedtime was defined as phone use from 9:00 pm to the time of sleep.”

11） Line 158: reference should be after period.

Reference has been moved after period.

12) Line 161-162: “There are 10 questions for positive and 10 for negative affects”, redundant.

This sentence was deleted according to the comment.

13) Line 172-173: “when the number presented is not identical to the previous one” replaced with “if not”.

This sentence was rephrased according to the comment.

14) Line 176-179: confused. Need rephrase.

Thank you for the suggestion. These sentences were rephrased as “There were six blocks (two for each condition), each block consisting of 30 trials. Each block lasted for approximately two minutes, leading to a total testing time of 12 minutes. The practice consisted of 10 trials for all conditions, and once all the practice trials were correctly completed, participants were allowed to start the task.”

15) Table 1 and 2: ugly, need revise.

Thank you for the suggestion. We have beautified the table and adjusted the page to be horizontal according to PLOS ONE's style requirements.

16) Line 202-246 of results: Significance for some tests cannot be found in Table 2, need correct for each. For example: Table 2: A star (*) is missing in the group effect of sleep latency and time effect of sleep duration according to Line 205 and 207-208, respectively.

Fig 2 and 3: Significance (* and **) should be noted with corresponding p-value for each test. Line 222-225: correct positive affect for significance in Table 2 and Figs.

Line 227: it is not significant for p=0.06. Correct Table 2 and Figs.

Line 212-219: correct Table 2 and Figs for significance.

Line 238-246: not corresponding to Table 2.

Thank you for your comments. I reply to these comments together, because they were all related with significance marks, which were similar to the major comments. The miss of significance marks in figures have been added. After re-check it, we think there was no missing marks in tables. Table 2 was the results of between-subject analyses of variance.

17) Line 233-234, Line 236-237: Don’t know it is for accuracy or reaction time, or for 2-back accuracy?

Thank you for the suggestion. These sentences have been rephrased to make it clearer.

18) Line 235: redundant with Line 232

There was a mistake of writing. “1-back accuracy” in line 235 was correct as “2-back accuracy”.

19）“In this” study, “the effects” …. Was investigated.

This sentence was rephrased according to the comment.

20）Line 268: Sleep quality “was” improved significantly.

This sentence was rephrased according to the comment.

21）Line 269: One research “group”

This sentence was rephrased according to the comment.

22）Line 270-271: adolescents “who” stopped, delete “and”

This sentence was rephrased according to the comment.

23）Line 276-277: arousal “was” decreased, suggesting that it…

This sentence was rephrased according to the comment.

24）Line 278: no published report has shown

“To our knowledge, no study has investigated the effect of restricting……” were corrected as “To our knowledge, no published report has shown the effect of restricting……”.

25）Line 281: It was reported that insomnia patients have somatic, cognitive and cortical arousal [21-24].

This sentence was rephrased according to the comment.

26）Line 282-283: “Content in the mobile…sleep quality” should be in Line 278.

“Content in the mobile….sleep quality” were moved to line 278 according to the comment.

27) Line 284-285: redundant. Sleep “plays”

Redundant sentences have been deleted according to the comment. “has” was corrected as “plays”.

28) Line 288-289: rephrase

These sentences were rephrased as “Previous studies report that the mood may be affected by sleep deprivation and sleep restriction [25-28], while sleep extension was found to reduce the tendency to go into a depressed mood [29]” were rephrase as “Previous studies have found the impact of sleep deprivation and sleep restriction on mood[26-29], and the influence of sleep extension on the reduction of the tendency to fall into a depressed mood [30].”

29) Line 294: suggesting

We think “suggest” was more appropriate. The sentence was “These results suggest that restricting mobile phone usage around bedtime could improve working memory.”

30）Line 296-300: rephrase

These sentences have been rephrased as “Pervious researches on children and adolescents have demonstrated that sleep restriction and deprivation could affect cognitive functions [30]. Increasing sleep duration could enhance cognitive performance [31]. In our study, sleep quality of individuals of the intervention group was shown to have improved significantly, which had a positive effect on cognitive function” were rephrase as “Previous studies on children and adolescents have demonstrated that sleep restriction and deprivation could affect cognitive functions [31]. Increasing sleep duration could enhance cognitive performance [32]. In our study, sleep quality of individuals in the intervention group was shown to have improved significantly, having a positive effect on cognitive function.”

31) Line 304-308: confused

Thank you for the suggestion. To make it clearer, line 304-308 were corrected as “Second, the participants of both groups agreed that excessive mobile phone use around bedtime could disturb their sleep and were all willing to reduce the amount of use around bedtime. However, these results might not be generally applicable to participants who did not agree that phone use around bedtime affects sleep and who lacked motivation to restrict mobile phone use around bedtime”.

32) Line 309: remove “four weeks of”, add “for four weeks” after “bedtime”.

This sentence was rephrased according to the comment.

**Reviewer #2:**

1) Authors should briefly provide a description/synonym of netizens

Thank you for your suggestion. Chinese netizen is someone who likes surfing, shopping, making friends online. It is called “Wang Min” in Chinese. “Chinese netizen” was corrected as “Chinese Internet users”，which is easier to be understood.

2) There are some typos. Some sentences begin with "And". English should be revised for the sake of clarity.

Thank you for your suggestion. We have double checked the language and made it clear carefully.

3) Authors did not follow reporting guidelines for randomized trials (for example, the title does not contain informative keywords). Did authors register the randomized trial in a database of RTs?

We are grateful for the suggestion. To be clearer and with the guidelines for randomized trials, we have added these informative keywords in title. We didn’t register the randomized trial in a database of RTs. This is a non-clinical randomized controlled study. Participants in this study were mainly students from our school who have some symptoms of insomnia, but not meet the diagnostic criteria for insomnia. Insomnia patients in clinical take sleep medicine, which have impact on cognitive function. According to the purpose of our experiment, we think the subject from non-clinical condition is suitable.

4) Sample size is very small. Seems (to my opinion) that the study is underpowered to make sufficiently strong statements and conclusions. Is it a pilot study? How much is the statistical power found (in tems of effect sizes)? And the post-hoc power Did authors perform a sample power analysis?

Thank you for your suggestion. As this was a pilot study primarily aimed at assessing feasibility and acceptability of the intervention, sample size was small and sample size calculations were not performed which limits the interpretation of the results. In future studies, we will further expand the sample size.

5) Some important studies are not mentioned (one for all: Mobile Phone Use and Mental Health. A Review of the Research That Takes a Psychological Perspective on Exposure. Thomée S. Int J Environ Res Public Health. 2018 Nov 29;15(12)).

Yes, we may have neglected to cite some important literature. We have added this study to our manuscript according to the comments. However, this article mainly talked about bedtime mobile phone use, so most of the literature cited was about the effect of "bedtime mobile phone use" on sleep.

6) Graphs miss bars of standard deviations.

Thank you for your suggestion. We have added bars of standard deviations in figures according to the comment.

We would also like to thank you for allowing us to resubmit a revised copy of the manuscript.

We hope that the revised manuscript is accepted for publication in the Journal of PLOS ONE.

Sincerely,

Jing-wen He e-mail: 784471783@qq.com

Zhi-hao Tu e-mail: 736466216@qq.com

Yun-xiang Tang e-mail: plostang@sina.com
